# Season of Birth and Risk for Adult Onset Glioma

**DOI:** 10.3390/ijerph7051913

**Published:** 2010-04-28

**Authors:** Jimmy T. Efird

**Affiliations:** Center for Health of Vulnerable Populations, Office of the Dean, School of Nursing, University of North Carolina, 237A McIver Building, Administrative Drive, Greensboro, NC 27402-6170, USA; E-Mail: jimmy.efird@stanfordalumni.org; Tel.: +1-650-248-8282

**Keywords:** gliomas, farming, allergies, handedness

## Abstract

Adult onset glioma is a rare cancer which occurs more frequently in Caucasians than African Americans, and in men than women. The etiology of this disease is largely unknown. Exposure to ionizing radiation is the only well established environmental risk factor, and this factor explains only a small percentage of cases. Several recent studies have reported an association between season of birth and glioma risk. This paper reviews the plausibility of evidence focusing on the seasonal interrelation of farming, allergies, viruses, vitamin D, diet, birth weight, and handedness. To date, a convincing explanation for the occurrence of adult gliomas decades after a seasonal exposure at birth remains elusive.

## 1. Introduction

Gliomas are a heterogeneous group of neoplasms in terms of cellular origin and location. They represent the most common malignant brain tumors [1]. Despite considerable efforts to unravel the etiologic basis for this cancer and attempts to find a cure, gliomas largely remain refractory to treatment. Except for a small percentage of cases, the tumors continue to show high morbidity and mortality. Established risk factors include ionizing radiation and a few genetic syndromes [2]. However, the attributable risk for these factors is small and can only explain a fraction of cases.

Evidence of an association between adult-onset glioma and date of birth suggests an etiologic role for a seasonally variable environmental exposure occurring prenatally around the time of birth. The period between an environmental exposure and the appearance of cancer may be lengthy, as illustrated by the development of vaginal cancer in daughters years after their mothers used diethylstilbestrol (DES) during pregnancy [3]. Similarly, exposure to asbestos 70 years prior to the development of mesothelioma [4], suggests a “one insult, co-carcinogenic model” of cancer development [5]. Risk factors that may exhibit seasonal epidemicity include exposure to infectious agents, allergens, pesticides, indoor environmental tobacco smoke, and other sources of polycyclic aromatic hydrocarbons (e.g., soot from fires for home heating), and use of antihistamines. Other putative candidates include harmonic variation in population mixing, diet, temperature, humidity, sunlight/photoperiod, levels of vitamin D3, and endogenous hormones [6,7].

The origins of many childhood and adult malignancies are believed to be determined during a finite perinatal window. During this period the brain and immune systems are less developed, undergoing greater cell growth and differentiation, and possibly more susceptible to DNA and epigenetic changes underlying cancer development in the near or distant future [5,8–13]. In this model, environmental exposures and lifestyle changes that occur afterwards play a mainly secondary role to the programming or imprinting that occurs during pregnancy and/or early infancy [14,15].

## 2. Methods

### 2.1. Search Strategy

Articles were identified by systematically searching the Medline/PubMed database from inception to January 2010 using the key words: brain tumors and season of birth. Furthermore, we manually examined the bibliography of the papers that were identified electronically. We also searched for unpublished studies, PhD dissertations, internal reports, and conference proceedings/abstracts. Finally, to make our search inclusive of those studies in which season of birth was a secondary factor we checked the tables of every article known to us that examined risk factors for brain tumors. A similar process was used to identify papers on risk factors related to season of birth.

### 2.2. Inclusion Criteria

Studies were included only if the target population, outcome and exposures of interest were clearly defined and results were based on valid statistical methodology with evaluation of errors and discussion of study bias. Studies in which participants were chosen at convenience were not considered in this review. In the case of a cohort appearing in the literature multiple times only the most recent version was included since it provided the largest number of cases and the longest follow-up. Priority was given to papers that presented original data rather than reviews or meta-analyses.

## 3. Review of Literature

Several recent studies have explored season of birth as a risk factor for adult glioma, but results have been mixed (Table 1). Three independent reports have observed a peak risk for winter births [16–18], while no seasonal variation for risk was noted in two other epidemiologic studies [19,20]. A multi-centered study of four Midwest states examining rural and farm exposures observed a statistically significant increased risk of adult gliomas for springtime births, but only among those born “off” a farm [21]. Similarly, the study of childhood brain tumors and season of birth have yielded equivocal results [22–26].

## 4. Risk Factors Related To Season of Birth

The underlying rationale for the occurrence of a glioma decades after a seasonal exposure remains elusive. No specific etiologic agent to date has been identified to support such a long induction period for this cancer. Several hypothetical arguments have been offered in explanation of a season of birth effect for adult gliomas, if such an association exists. Thus far, the major evidence in support of a seasonal effect involves farming, allergies/immunity, viruses, and vitamin D. Other possible factors related to seasonal birth are diet, birth weight, and handedness.

### 4.1. Farming

Farming, by virtue of its seasonal nature, conveys increased risk at certain times of the year of exposure to pesticides and other agricultural chemicals. Insecticides and herbicides constitute the bulk of farm chemical exposures: two key routes of exposure are dermal and respiratory [27], bypassing detoxification in the liver. Many farm chemicals are classified as probable or likely human carcinogens by the US Environmental Protection Agency (EPA), most notably acephate, dichlorvos, dimethoate, lindane, parathion, phosmet, and tetrachlorvinphos [28].

Indoor exposures to these agents frequently occur [29,30]. Parents and other individuals track dust and dirt into the home, on shoes and clothes, after working on the farm. Household dust concentrations of azinphosmethyl, chlorpyrifos, parathion, and phosmet have been found to be significantly higher in farmer or farmworker homes than in referent homes [29]. Similarly, indoor-outdoor pets are efficient carriers of agents into the home, and also pose an exposure source for pesticides from anti flea/tick collars, sprays, and shampoos. Homes with high pet activities have been shown to have greater levels of pesticide residues in sampled carpet dust than homes with low pet activities [31,32]. Airborne farm pollutants also may enter the home environment by way of the wind, after spraying, gassing, or open-air application. Newborn and infant children have greater contact with these contaminants since they lie, crawl and play near the ground and frequently place their hands, objects or food in their mouths [29,33,34].

In tandem with living on a farm, infants have unique vulnerabilities that may lead to higher levels of exposures in the same environment than adults [9]. For example, their skin surface area is comparatively larger and more permeable to lipophilic compounds, which serves to maximize the absorption of compounds such as pesticides into the bloodstream [35–37]. Dryness, rashes, and related skin conditions, to which infants may be particularly susceptible, serve to disrupt the protective barrier of the skin [36,38]. Once in the circulatory system, compounds enter the brain of the infant relatively quickly as the blood-brain barrier is not fully operational until at least 6 months of age [10].

At birth, children are less able to detoxify chemicals and other inorganic substances commonly used on farms [33,34], and they tend to have higher concentrations in their blood due to a decreased capacity to bind plasma protein [35]. They drink more water and eat more foods per unit of body weight than adults and will therefore ingest more toxins from well water and agriculturally treated foods [33,34]. Furthermore, they are more sensitive to airborne chemicals since their resting air intake is about twice that of an adult [34]. The commonly used organophospate pesticide chlorpyrifos (CPF) is known to specifically target the immature brain, from the early embryonic stage to the postweaning period, and it causes a host of irreversible changes to developing brain cells [39]. (Pesticides also are used outside the farm in urban schools, homes, and day-care centers for control of roaches, rats, and other vermin, and conceivably their peak period of application may differ seasonally from that on farms [33,40].)

While farmers live longer than the population-at-large [41], and die less frequently from cancer in general [42], several epidemiologic studies have found an increased risk for brain cancer in this group [43–50]. Farmers represent a diverse group (e.g., with dairy, field crop, hog, beef cattle, poultry, fish, marijuana, cotton, and organic), and the heterogeneity of brain cancer risk for farmers could reflect differences in activities, as well as in the type, magnitude, and seasonality of exposures.

### 4.2. Allergies and Immunity

The perinatal period represents a time of heightened sensitivity to environmental allergens [51,52]. Within this window the peak for immune system priming or susceptibility is believed to vary differentially in response to presenting antigens [53,54]. Contact with allergens during early childhood may enhance the risk of developing allergies later in life, especially in individuals with a predisposition for atopic and allergic conditions [51,55].

Efforts to reduce allergen exposure immediately after birth often only delay the onset of allergic conditions, rather than preventing it, suggesting that immunologic memory may predate birth [15]. While genetics constitute a key determinant of immunologic memory and immune system maturation [56–58], environmental factors also are believed to play an important role in the development of allergies, as illustrated by the discordance of allergies in twin studies [59], the increasing prevalence of atopic diseases over time [15,60], and the different incidence of allergies in immigrant and country of origin populations [61].

In a number of studies, season of birth has been associated with the development of allergic conditions later in life, such as asthma [61–65], atopic dermatitis [66], house dust mite allergy [60,61,65,67–69], cockroach allergy [70,71], grass/birch/hay and other pollen allergy [51,60,63,67, 68,72–76], animal dander allergy [51,67], mold allergy [74], food allergy [51,55,67], allergic rhinitis [62,77], and otherwise unspecified atopy (NOS) [55,78]. In the case of cockroach sensitivity, over 60% of inner-city residents with asthma test positive for cockroach allergen [79], with black children and those receiving public assistance most likely to be sensitized [70]. The rate of cockroach sensitization at the age of 5 years was higher for winter births (72%), than for spring (48%), summer (42%) and fall (43%) births [70].

Factors known to influence the development of asthma include race [80,81], low yearly family income [81], less than a high school education [81], residence in high poverty areas [81], exposure to urban air pollution [64] and childhood viral infections [64,82]. A significant percent of infants with a history of respiratory syncytial virus (RSV) bronchiolitis infection, which peaks during the winter season, develop asthma [64].

Asthma, allergies, and other atopic diseases have been inversely associated with adult glioma risk in several epidemiologic studies and meta-analyses [83–93], although some reports do not support this conclusion [94–98]. The exact biologic mechanisms underlying a protective effect are not clearly understood. It has been suggested that the inverse association may reflect increased immune surveillance on the part of the atopic individual [91,93]. Glioma patients are known to suffer from an impaired immune system [99], and the reduced association with allergies alternatively could be due to immunosuppression induced by the tumor (*i.e*., reverse causality) [84]. Inflammatory responses also may favor tumor development [93]. In animal studies, therapeutic immunity to intracranial tumors has been induced by peripheral immunization with interleukin-4 (IL-4) transduced glioma cells [100,101].

Being raised on a farm [102–114] or in a rural area [115] has been shown in some studies to protect against asthma, hay fever, and atopic sensitization. Although farm children are exposed to higher concentrations of airborne allergens, paradoxically they become sensitized less often and manifest a weaker sensitization response than non-farm controls [113]. Some researchers have hypothesized that the protective effect may be attributed to a form of “tolerance”. Such tolerance could conceivably develop early in life, following repeated exposure to high levels of allergens such as organic dusts, fungi, and endotoxins; this is perhaps the result of component lipopolysaccharides that excite Th1 responses and suppress the development of immunoglobulin-E (IgE)-antibodies [109,116].

Little is known about specific determinants of asthma and atopy in the farm setting. Any relationship with glioma risk is probably complex and must be interpreted in light of differences in farming practices and the protective ability of farming environments, especially with respect to microbial exposures [102,103,114]. While farmers and their families have greater contact with seasonal elements, they may be less prone to “sick building syndrome” than residents of urban areas [117]. The latter tend to live and work in a more closed indoor environment and thus suffer greater exposure to indoor pollutants (e.g., polychlorinated biphenyls, phthalates, phenols, flame retardants) [29]. Other mitigating factors may include a greater intake of saturated fat in the form of milk and butter, as well as a greater number of older siblings living at home in rural/farm communities [116]. Furthermore, farmers usually are non-smokers and healthier than the population-at-large [43,116,118–120]. By self-selection, those who manifest seasonal allergies may choose a career path other than farming (i.e., healthy worker effect) [116].

Differences in the definition of atopy, a lack of objective measures of atopy, residual confounding, and bias (e.g., detection, selection, interview, recall) are important to consider when interpreting the above studies [92,121]. Furthermore, regarding the role of allergies and immunity, there is no clear trend toward a decreasing risk for glioma with younger ages at onset of the allergic condition [121].

Interestingly, increased risk for glioma has been observed in patients with AIDS-related immuno-suppression [122–124], but not in those with iatrogenic immuno-suppression [125].

### 4.3. Viruses

Environmental exposures to viruses, microbes and other pathogens generally tend to occur more often in particular seasons [25,126,127]. Polyomaviruses have been shown to induce brain tumors in animals [128–131] and have been detected in human gliomas [132]. Other neuro-oncogenic viruses implicated in gliomas under laboratory conditions include human adenovirus type 12 and Rous sarcoma virus [133]. Some tumor viruses must be injected in animals on the first day of life to be effective, although they may not cause cancer until years later [7].

Epidemiologic evidence in support of a viral or pathogenic etiology for adult brain tumors remains controversial and the windows of interest of these studies have been outside the perinatal period. In adults, *Toxoplasma gondii* infection has been associated with a greater prevalence of astrocytomas [134], while decreased glioma risk has been associated with a history of infections/colds [88], and chicken pox [135,136]. On the other hand, increased risk for childhood brain tumors has been associated in some studies with a history of chicken pox [137], influenza [138,139], measles [138], general viral infections [140,141], and neonatal urinary tract infections [141]. In particular, a 7.5-fold OR (95% CI = 1.3–44.9) for low grade astrocytoma has been observed for neonatal urinary tract infections [141]. Bacterial urinary tract infections have been shown to follow a well defined seasonal pattern with a peak in August [127]. Similarly, seasonal occurrence has been observed for chicken pox, measles, influenza and many other common childhood infectious diseases [7,142].

A seasonal infectious etiology for disease is complicated by many factors [126]. The same infectious agent may present a different pattern of incidence depending on the host location. A seasonal peak evident in the general population may not behave uniformly within certain subpopulations. Temperature, humidity, precipitation, and indoor air quality are among the mitigating factors that may affect the survival and transmissibility of a pathogen and explain different seasonal peaks for a disease in different studies. Other factors include poor nutrition, crowding, seasonal travel, hygiene practices, cultural practices in food consumption/preparation, changes in herd immunity, or evolution of the infectious agent over time. Furthermore, seasonal variation in immune function may increase host susceptibility to infections at certain times of the year [143,144]. Given the rarity of some infections and brain tumors, the interpretability of seasonal patterns, or the lack thereof, may be confounded by time- and/or space-dependent factors [126].

### 4.4. Vitamin D

Vitamin D is a fat-soluble prohormone which operates via a nuclear receptor [145]. Maternal serum vitamin D levels tend to be closely correlated with infant cord serum levels [146,147]. This light synthesized steroid, resulting from the absorption of ultraviolet B photons by 7-dehydrocholesterol in the skin [148], has been suggested as a possible factor underlying the season of birth risk for adult gliomas observed in some studies [145]. Levels of vitamin D are known to fluctuate depending on sunlight exposure, which is highest in the summer for the northern hemisphere and tends to increase for latitudes nearer the equator [145,148–150].

Antiproliferative and proapoptotic activity of vitamin D3 has been noted in model glioma cell lines and a primary culture, as well as tumor tissue experiments [145]. Further, prenatal vitamin D3 depletion has been associated with altered brain development in rodent studies [151]. Vitamin D receptors are present in most cells and tissues in the body [152], including the brain [148], so this hormone is believed to play an important role in disease management and cancer prevention as a modulator of immune system activity [148,153–155].

Children who live in polluted, inner city environments and who are deprived of regular sunlight exposure have been shown to suffer a higher prevalence of rickets (due to vitamin D deficiency) than children in rural areas [148]. Seasonal variation in vitamin D levels also has been suggested as a factor differentially affecting newborn growth plate width, and this may explain why babies born in Australia around October (spring) are relatively heavier than those born at other times [156].

### 4.5. Diet

The “developmental plasticity hypothesis” refers to the critical developmental period *in utero* when the fetus is plastic and adaptively imprints to its environment. *In utero* adaptation may lead to lifelong changes in the body’s metabolism, hormone regulation, and the number of cells present in key organs [14,157,158]. If this is the case, adult health outcomes may reflect season of birth if food intake is seasonal, and if exposure occurs at a critical stage of fetal development. Certain foods and the chemicals contained in these foods may be more or less available at particular times of the year due to crop cycles, storage capability, transportation access, and cost; and the use of preservatives in foods also may vary accordingly. N-nitroso compounds are a potent inducer of cancer and brain tumors in various animal species [159,160], and fetal exposure produces more tumors than postnatal exposure [161]. Foods high in N-nitroso compounds have been associated with an increased risk for brain tumors in some studies [162–165]. These compounds may be present in foods through their use as preservatives or in fertilizers [166].

Vitamin C, which is mostly found in seasonal fruits and vegetables, can react with and then inhibit nitrite formation of nitrosamines [163]. An 18-fold greater risk of primary adult brain tumors has been found among individuals who consumed large amounts of processed meats (high in N-nitroso content) and drank orange/grapefruit juice once per week or less, than among those who consumed less processed meat and drank orange/grapefruit juice more often [164]. However, a recent multi-centered international case-control study of adult diet and brain tumor risk found no evidence of confounding or effect modification by vitamin supplementation, especially in the association between consumption of cured meats (containing high levels of nitrates) and gliomas [167].

### 4.6. Birth Weight

Birth weight has been reported to vary by season of birth [168–170]. High birth weight is an important predictor of adult obesity [171], and a higher body mass index (BMI) has been linked to greater risk for adult-onset glioma [172]. In children, high birth weight has been associated with increased risk for astrocytoma and medulloblastoma/primitive neuroectodermal tumors (PNET) [173]. Temporal patterns in birth weight may result from seasonal variations in food availability. For example, birth weights in agricultural societies are highest at the peak of the dry season, corresponding to the time of harvesting and greatest food availability [174]. Interestingly, very low birth weight has been associated with left- or mix-handedness [175,176], a factor associated in some studies with season of birth [177] and reduced glioma risk [178].

### 4.7. Handedness

Handedness is believed to be determined *in utero* [179], possibly through hormonal influences [180–183], and has been linked with immune disease [184,185], various developmental learning disorders [184], epilepsy [186], and breast cancer risk in both pre- and post-menopausal women [187–190]. Exposure to a high level of testosterone during the earlier part of pregnancy is thought to promote right brain development and influence hand preference, although the evidence in support of a relationship is not conclusive [191]. Testosterone, progesterone, oestradiol, and lutenising hormone levels in women have been reported to be higher in spring than in autumn [192–194].

Results from several studies hint that non-genetic factors may play an important role in the determination of handedness. Women exposed *in utero* to the estrogen-like compound DES show a higher prevalence for left handedness [195,196]. Similarly, left-handedness has been linked to congenital adrenal hyperplasia, a condition characterized by high *in utero* levels of androgeizing hormones [197]. Of note, brain tumors are known to contain sex steroid receptors (e.g., estrogen, androgen, progestin) [198], and sex hormones have been shown to promote brain tumors in animal studies [199,200]. Mice reared under conditions favoring the right limb develop a right-limb preference, and vice versa [201], further suggesting that environmental factors influence handedness. Additionally, the discordance of handedness in twin studies illustrates that genetics alone cannot explain the development of handedness [202–207].

Handedness has been shown to follow a gender-invariant seasonal pattern, with more left-handers than right-handers born between March and July [177,178]. Being right-handed has been associated with a greater risk for glioma than being left-handed or ambidextrous [178]. However, for left-handed and ambidextrous participants born from late fall through early spring, glioma risk also has been shown to be higher than for those born at other times of the year [16]. A study of 15,390 Brazilian students was unable to detect a difference in the season of birth between right- and left-handers. However, little seasonal variation exists in the light/dark cycle in the area where the majority of study participants were born [208].

The putative connection between handedness, season of birth, and glioma risk remains difficult to interpret. For example, glioma risk is greater for males than females [2], yet the pattern of seasonal influence upon handedness does not vary significantly between females and males [177]. A higher incidence of sinistrality has been observed for individuals born in winter and autumn than for those born summer and spring [191]. The incidence of left-handedness is disproportionately higher among males [202], a finding which is consistently observed across species [201,209–212]. Risk for glioma also is nearly equal for left-handed and ambidextrous individuals but higher for right-handers. Overall, handedness appears to be a surrogate marker for an underlying but unidentified risk factor that interacts with seasonality and other factors to possibly explain glioma risk.

## 5. Discussion

Season of birth in its own right cannot be deemed a cause for glioma. Rather, it is a surrogate marker for underlying risk factors that may offer subtle clues about the possible perinatal etiology of adult gliomas.

There is probably no specific etiologic agent acting in isolation that completely explains a season of birth association with adult glioma risk. If an association exists the effect probably represents the interplay between various environmental, genetic, lifestyle, and socioeconomic factors, operating on mother and child within a precise window of vulnerability. The mechanism of action may involve epigenetic processes or DNA changes with the capacity to modulate or influence oncogenic outcomes in the distant future. A patient’s innate and adaptive immune response and a host of hormonal and metabolic factors, likely play important and closely interrelated roles in understanding the complex biologic and etiologic mechanisms in the season of birth effect for adult gliomas.

In the studies conducted to date, risk estimates and p-values for the association between season of birth and glioma risk have been nominal to borderline in strength. However, a weak association does not necessarily imply the absence of causality, nor is a strong association necessary or sufficient for causality [213,214]. The ability to find associations between specific seasonal risk factors occurring around the time of birth and adult glioma incidence is limited by the rarity of this cancer in the population and the vast time period from exposure to disease. While the risk factors outlined in this review do not conflict with our current understanding of the natural history of cancer or glioma specifically, the evidence in support of a causal relationship has not been consistent. To some degree, effect modification or publication bias may explain the inconsistencies. At minimum, if a causal relationship exists, season of birth meets the criteria of temporality as the period surrounding birth clearly predates disease occurrence. However, the exact window of exposure when the underlying risk factor(s) most occur to cause disease is unknown.

On the present evidence, it is not possible to definitively answer the question of how season of birth influences glioma risk, or to favor any specific hypothesis. Many questions remain and future research will benefit from focused studies aimed at specific exposures and subpopulations, allowing for more detailed analyses by histologic subtypes.

## Figures and Tables

**Table 1.** Summary of studies on season of birth and adult glioma risk.

**Reference, Year, Country**	**Study design**	**Cases (M=Male, F=Female)**	**Referents**	**Risk estimates and/or p-values**	**Comments**
Brenner, *et al.* 2004 U.S.A [16]	Case-referent	Low-grade glioma, n = 135. High-grade glioma, n = 354. 63% of the low-grade gliomas were oligo-dendrogioma, astrocytoma, or mixed gliomas. 86% of high-grade gliomas were glioblastoma or anaplastic astrocytoma. M/F ratio = 1.3. Mean age = 52yrs.	Frequency matched (by hospital, age, sex, race, ethnicity, and distance of residence from hospital), n = 799.	Sinusoidal test of periodicity, χ^2^=6.1 (2df), p = 0.04. Risk peaked in February and troughed in August (OR = 1.5, CI not provided).	Incident cases. Hospital-based referents. Adjustment by education, marital status, place of birth, handedness, birth order, and history of allergy or auto immune disease did not change results. Excluding different diagnostic subgroups of referents from the analysis did not materially change the estimated parameters of the periodic function.
Efird 2009 U.S.A. [21]	Case-referent	Primary intracranial gliomas, n = 797.	Frequency matched within state of current residence to cases (2:1 to projected number of cases) by age and sex, n = 1,174.	Sinusoidal logistic regression model. All participants, peak day = 169, p = 0.0469. Born “on” farm, peak day = 283, p = 0.1579. Born “off” farm, peak day = 150, p = 0.0049. GBM, peak day = 146, p = 0.1456. Non-GBM, peak day = 187, p = 0.0603.	Adjusted for age, sex, and state of residence. Population-based controls. Participants were not asked about handedness.
Houben, *et al.* 2005 The Netherlands [20]	Tumor registry cases to all cancer patients comparison.	Pilocytic astrocytoma, n = 37. Other astrocytoma, n = 1,064. Oligodendroglioma, n = 131. Other glioma, n = 234. 59.5% males. Median age = 52yrs. Age range = 0–92yrs.	Monthly births adjusted to all cancer patients registered by the cancer registry.	Edward’s test for sinusoidal variation, p < 0.05.	Hospital-based referent population. Adults analyzed together with children.
Koch, *et al.* 2006 Germany [17]	Tumor registry to population cases comparison.	Glioblastoma, n = 299M, 202F. Mean age (±SD) = 57.1 ± 14.2 yrs.	Population normalized monthly births	Circannual cosinor model, r^2^ = 0.34, p < 0.05. Maximum frequency of births was found in Jan. Monthly mean birth freq = 127.9 (SEM = 7.1).	Analysis not adjusted for age, sex. Did not specify the reference years of the birth rates used for normalization of the observed incidence data.
Mainio, *et al.* 2006 Finland [18]	Surgical record cases to population census comparison.	Grade I–II gioma, n = 9M, 10F. Grade III–IV glioma, n = 14M, 8F. Meningioma, n = 7M, 26F. Pituitary adenoma, n = 5M, 3F. Acoustic neurinoma, n = 4M, 3F. Other, n = 0M, 6F. Mean age (±SD), F = 49.4 ± 11.5 yrs,. M = 49.4 ± 11.5 yrs.	Monthly births in the general population over the years corresponding to the entire range of year of births of the brain tumor patients (born in the years 1915–1971).	Observed to expected ratio = 1.3 (95%CI = 1.01–1.77). Comparison: December–February to March–November. Winter trough of births reported for low-grade glioma (adj. ratio = 0.4, CI not provided).	Risk only separately reported for low-grade gliomas. Excluded cases not surgically treated.
Staykov, *et al.* 2009 Germany [19]	Tumor registry cases to population census comparison.	Mixed glioma, n = 46M, 32F. Ependymoma, n = 48M, 51F. Other glioma, n = 34M, 17F. Anaplastic astrocytoma, n = 122M, 99F. Pilocytic astrocytoma, 15M, 8F. Other astrocytoma, n = 138M, 97F. Glioblastoma, 805M, 560F. Oligodendroglioma, 52M, 50F. Mean age (±SD), M = 54 ± 14, F = 54 ± 14.	Monthly births for the years 1931 to 1986 (except 1944) in the general Bavarian population.	Roger-test of seasonality, p = 0.54 F, p = 0.11 M. The estimated peak-trough ratio of glioma in persons born in the month with the greatest number of cases compared to month with lowest number was 1.07 in M (95%CI=1.00–1.25) and 1.16 in F (95%CI=1.00–1.39).	Incident cases. Data not analyzed separately by low- and high-grade glioma. The analysis of time trends in the distribution of monthly births adjusted for length of month revealed a pronounced absolute maximum of monthly births in February and March prior to 1965, which has gradually decreased in prominence during the subsequent decades. Analysis not adjusted for handedness, education level, or history of allergy/autoimmune disease.

## References

[1] CBTRUS Statistical Report Primary Brain Tumors Diagnosed in the United States in 2004–2005 Central Brain Tumor Registry of the United States Hinsdale, IL, USA 2009

[2] Wrensch M Minn Y Chew T Bondy M Berger M Epidemiology of primary brain tumors: Current concepts and review of the literature Neuro. Oncol 2002 4 278 299 12356358 10.1093/neuonc/4.4.278 PMC1920665

[3] Herbst A Ulfelder H Poskanzer D Adenocarcinoma of the vagina: association of maternal stilbestrol therapy with tumor appearance in young women N. Engl. J. Med 1971 284 878 881 5549830 10.1056/NEJM197104222841604

[4] McDonald A McDonald J Epidemiologic surveillance of mesothelioma in Canada Can. Med. Assoc. J 1973 109 359 362 4742473 PMC1946869

[5] Polednak A Latency periods in neoplastic diseases Am. J. Epidemiol 1974 100 354 356 4422307 10.1093/oxfordjournals.aje.a112045

[6] Efird J Nielsen S A method to model season of birth as a surrogate environmental risk factor for disease Int. J. Environ. Res. Public Health 2008 5 49 53 18441405 10.3390/ijerph5010049 PMC3684403

[7] Bailar J Gurian J Month of birth and cancer mortality J. Natl. Cancer Inst 1964 33 237 242 14207843

[8] Rice J Ward J Age dependence of susceptibility to carcinogenesis in the nervous system Ann. N.Y. Acad. Sci 1982 381 274 289 6953795 10.1111/j.1749-6632.1982.tb50392.x

[9] Selevan S Kimmel C Mendola P Identifying critical windows of exposure for children’s health Environ. Health Perspect 2000 108 451 455 10852844 10.1289/ehp.00108s3451 PMC1637810

[10] Rodier P Developing brain as a target of toxicity Environ. Health Perspect 1995 103 73 76 8549496 10.1289/ehp.95103s673 PMC1518932

[11] Rodier P Vulnerable periods and processes during central nervous system development Environ. Health Perspect 1994 102 121 124 7925182 10.1289/ehp.94102121 PMC1567093

[12] Feinberg A Ohlsson R Henikoff S The epigenetic progenitor origin of human cancer Nat. Rev. Genet 2006 7 21 33 16369569 10.1038/nrg1748

[13] Lund A Lohuizen M Epigenetics and cancer Genes Dev 2004 18 2315 2335 15466484 10.1101/gad.1232504

[14] Barker D The developmental origins of adult disease Eur. J. Epidemiol 2003 18 733 736 12974544 10.1023/a:1025388901248

[15] Warner J Jones C Jones A Warner J Prenatal origins of allergic disease J. Allergy Clin. Immunol 2000 105 S493 S496 10669530 10.1016/s0091-6749(00)90049-6

[16] Brenner A Linet M Shapiro W Selker R Fine H Black P Inskip P Season of birth and risk of brain tumors in adults Neurology 2004 63 276 281 15277620 10.1212/01.wnl.0000129984.01327.9d

[17] Koch H Klinkhammer-Schalke M Hofstädter F Bogdahn U Hau P Seasonal patterns of births in patients with glioblastoma Chronobiol. Int 2006 23 1047 1052 17050217 10.1080/07420520600921088

[18] Mainio A Hakko H Koivukangas J Niemelä A Räsänen P Winter birth in association with a risk of brain tumor among a Finnish patient population Neuroepidemiology 2006 27 57 60 16847388 10.1159/000094578

[19] Staykov D Radespiel-Tröger M Meyer M Petsch S Schwab S Handschu R Birth month and risk of glioma in adults: a registry-based study in Bavaria Chronobiol. Int 2009 26 282 292 19212841 10.1080/07420520902761778

[20] Houben M Coebergh J Birch J Tijssen C van Duijn C McNally R Space-time clustering patterns of gliomas in the Netherlands suggest an infectious aetiology Eur. J. Cancer 2005 41 2917 2923 16274986 10.1016/j.ejca.2005.06.028

[21] Efird J Season of birth and risk for adult gliomas (abstract) Brain Tumor Epidemiology Consortium Annual Meeting Abstract Session The Houstonian Hotel, Houston, Texas 4–6 April, 2009

[22] McNally R Cairns D Eden O Alexander F Taylor G Kelsey G Birch J An infectious aetiology for childhood brain tumours? Evidence from space-time clustering and seasonality analyses Br. J. Cancer 2002 86 1070 1077 11953851 10.1038/sj.bjc.6600228 PMC2364189

[23] Heuch J Heuch I Akslen L Kvåle G Risk of primary childhood brain tumors related to birth characteristics: a Norwegian prospective study Int. J. Cancer 1998 77 498 503 9679748 10.1002/(sici)1097-0215(19980812)77:4<498::aid-ijc4>3.0.co;2-p

[24] Yamakawa Y Fukui M Kinoshita K Ohgami S Kitamura K Seasonal variation in incidence of cerebellar medulloblastoma by month of birth Fukuoka Igaku Zasshi 1979 70 295 300 478422

[25] Hoffman S Schellinger K Propp J McCarthy B Campbell R Davis F Seasonal variation in incidence of pediatric medulloblastoma in the United States, 1995–2001 Neuroepidemiology 2007 29 89 95 17925600 10.1159/000109502

[26] Halperin E Miranda M Watson D George S Stanberry M Medulloblastoma and birth date: evaluation of 3 U.S. datasets Arch. Environ. Health 2004 59 26 30 16053206 10.3200/AEOH.59.1.26-30

[27] Nielson J Nielson F Sørensen J *In vitro* percutaneous penetration of five pesticides—effects of molecular weight and solubility characteristics Ann. Occup. Hyg 2004 48 697 705 15509631 10.1093/annhyg/meh070

[28] Chemicals evaluated for carcinogenic potential Office of Pesticide Programs, Health Effects Division, US Environmental Protection Agency Washington, DC, USAApril 2003.

[29] Butte W Heinzow B Pollutants in house dust as indicators of indoor contamination Rev. Environ. Contam. Toxicol 2002 175 1 46 12206053

[30] Starr H Aldrich F MacDougall W Mounce L Contribution of household dust to the human exposure to pesticides Pestic. Monit. J 1974 8 209 212 4142653

[31] Morgan M Stout D Wilson N Feasibility study of the potential for human exposure to pet-borne diazinon residues following lawn applications Bull. Environ. Contam. Toxicol 2001 66 295 300 11178642 10.1007/s001280004

[32] Nishioka M Burkholder H Brinkman M Lewis R Distribution of 2,4-Dichlorophenoxyacetic acid in floor dust throughout homes following homeowner and commercial lawn applications: quantitative effects of children, pets, and shoes Environ. Sci. Technol 1999 33 1359 1365

[33] Landrigan P Claudio L Markowitz S Berkowitz G Brenner B Romero H Wetmur J Matte T Gore A Godbold J Wolff M Pesticides and inner-city children: exposures, risks, and prevention Environ. Health Perspect 1999 107 431 437 10346991 10.1289/ehp.99107s3431 PMC1566233

[34] Landrigan P Risk assessment for children and other sensitive populations Ann. N.Y. Acad. Sci 1999 895 1 9 10676405 10.1111/j.1749-6632.1999.tb08073.x

[35] West D Worobec S Solomon L Pharmacology and toxicology of infant skin J. Invest. Dermatol 1981 76 147 150 7240782 10.1111/1523-1747.ep12525553

[36] Fenske R Black K Elkner K Lee C Methner M Soto R Potential exposure and health risks of infants following indoor residential pesticide applications Am. J. Public Health 1990 80 689 693 1693041 10.2105/ajph.80.6.689 PMC1404741

[37] Wester R Maibach H *In vivo* percutaneous absorption and decontamination of pesticides in humans J. Toxicol. Environ. Health 1985 16 25 37 4068054 10.1080/15287398509530716

[38] Nielsen J Percutaneous penetration through slightly damaged skin Arch. Dermatol. Res 2005 296 560 567 15834614 10.1007/s00403-005-0555-y

[39] Garcia S Seilder F Slotkin T Developmental neurotoxicity elicited by prenatal or postnatal chlorpyrifos exposure: effects on neurospecific proteins indicate changing vulnerabilities Environ. Health Perspect 2003 111 297 303 12611658 10.1289/ehp.5791 PMC1241386

[40] Adgate J Kukowski A Stroebel C Shubat P Morrell S Quackenboss J Whitmore R Sexton K Pesticide storage and use patterns in Minnesota households with children J. Expo. Anal. Environ. Epidemiol 2000 10 159 167 10791597 10.1038/sj.jea.7500078

[41] Alavanja M Sandler D McMaster S Zahm S McDonnell C Lynch C Pennybacker M Rothman N Dosemeci M Bond A Blair A The Agricultural Health Study Environ. Health Perspect 1996 104 362 369 8732939 10.1289/ehp.96104362 PMC1469343

[42] Blair A Dosemeci M Heineman E Cancer and other causes of death among male and female farmers from twenty-three states Am. J. Ind. Med 1993 23 729 742 8506851 10.1002/ajim.4700230507

[43] Kristensen P Andersen A Irgens L Laake P Bye A Incidence and risk factors of cancer among men and women in Norwegian agriculture Scand. J. Work Environ. Health 1996 22 14 26 8685669 10.5271/sjweh.104

[44] Reif J Pearce N Fraser J Occupational risks for brain cancer: a New Zealand cancer registry-based study J. Occup. Med 1989 31 863 867 2607385 10.1097/00043764-198910000-00015

[45] Wingren G Axelson O Cluster of brain cancers spuriously suggesting occupational risk among glassworkers Scand. J. Work Environ. Health 1992 18 85 89 1604277 10.5271/sjweh.1597

[46] Ahlbom A Navier I Norell S Olin R Spännare R Nonoccupational risk indicators for astrocytomas in adults Am. J. Epidemiol 1986 124 334 337 3728449 10.1093/oxfordjournals.aje.a114393

[47] Musicco M Filippini G Bordo B Melotto A Morello G Berrino F Gliomas and occupational exposure to carcinogens: case-control study Am. J. Epidemiol 1982 116 782 790 7148804 10.1093/oxfordjournals.aje.a113468

[48] Musicco M Sant M Molinari S Filippini G Gatta G Berrino F A case-control study of brain gliomas and occupational exposure to chemical carcinogens: the risk to farmers Am. J. Epidemiol 1988 128 778 785 3421243 10.1093/oxfordjournals.aje.a115031

[49] Brownson R Reif J Chang J Davis J An analysis of occupational risks for brain cancer Am. J. Public Health 1990 80 169 172 2297060 10.2105/ajph.80.2.169 PMC1404624

[50] Heineman E Gao Y Dosemeci M McLaughlin J Occupational risk factors for brain tumors among women in Shanghai, China J. Occup. Environ. Med 1995 37 288 293 7796195 10.1097/00043764-199503000-00003

[51] Nilsson L Björkstén B Hattevig G Kjellman B Sigurs N Kjellman N Season of birth as predictor of atopic manifestations Arch. Dis. Child 1997 76 341 344 9166028 10.1136/adc.76.4.341 PMC1717161

[52] Warner J Jones C Jones A Miles E Francis T Warner J Immune responses during pregnancy and the development of allergic disease Pediatr. Allergy Immunol 1997 8 5 10 9455773

[53] Van Duren-Schmidt K Pichler J Ebner C Bartmann P Förster E Urbanek R Szépfalusi Z Prenatal contact with inhalant allergens Pediatr. Res 1997 41 128 131 8979301 10.1203/00006450-199701000-00020

[54] Jones A Miles E Warner J Colwell B Bryant T Warner J Fetal peripheral blood mononuclear cell proliferative responses to mitogenic and allergenic stimuli during gestation Pediatr. Allergy Immunol 1996 7 109 116 9116874 10.1111/j.1399-3038.1996.tb00117.x

[55] Nilsson L Kjellman N Atopy and season of birth Allergy 1996 51 138 139 8738526 10.1111/j.1398-9995.1996.tb04576.x

[56] Dold S Wjst M von Mutius E Reitmeir P Stiepel E Genetic risk for asthma, allergic rhinitis, and atopic dermatitis Arch. Dis. Child 1992 67 1018 1022 1520004 10.1136/adc.67.8.1018 PMC1793604

[57] Küster W Petersen M Christophers E Goos M Sterry W A family study of atopic dermatitis: clinical and genetic characteristics of 188 patients and 2,151 family members Arch. Dermatol. Res 1990 282 98 102 2353830 10.1007/BF00493466

[58] Ober C Hoffjan S Asthma genetics 2006: the long and winding road to gene discovery Genes Immun 2006 7 95 100 16395390 10.1038/sj.gene.6364284

[59] Falliers C de Cardoso R Bane H Coffey R Middleton E Discordant allergic manifestations in monozygotic twins: genetic identity versus clinical, physiologic, and biochemical differences J. Allergy 1971 47 207 219 4252251 10.1016/s0091-6749(71)80466-9

[60] Businco L Cantani A Farinella F Businco E Month of birth and grass pollen or mite sensitization in children with respiratory allergy: a significant relationship Clin. Allergy 1988 18 269 274 3396196 10.1111/j.1365-2222.1988.tb02869.x

[61] Smith J Springett V Atopic disease and month of birth Clin. Allergy 1979 9 153 157 445784 10.1111/j.1365-2222.1979.tb01536.x

[62] Åberg N Birth season variation in asthma and allergic rhinitis Clin. Exp. Allergy 1989 19 643 648 2598104 10.1111/j.1365-2222.1989.tb02761.x

[63] Wjst M Dold S Reitmeir P Stiepel E von Mutius E Month of birth and allergic disease at the age of 10 Clin. Exp. Allergy 1992 22 1026 1031 1468028 10.1111/j.1365-2222.1992.tb03032.x

[64] Gazala E Ron-Feldman V Alterman M Kama S Novack L The association between birth season and future development of childhood asthma Pediatr. Pulmonol 2006 41 1125 1128 17034060 10.1002/ppul.20442

[65] Warner J Price J House dust mite sensitivity in childhood asthma Arch. Dis. Child 1978 53 710 713 718239 10.1136/adc.53.9.710 PMC1545128

[66] Lewy H Meirson H Larson Z Seasonality of birth month of children with celiac disease differs from that in the General population and between sexes and is linked to family history and environmental factors J. Pediatr. Gastroenterol. Nutr 2009 48 181 185 19179880 10.1097/MPG.0b013e3181709530

[67] Aalberse R Nieuwenhuys E Hey M Stapel S ‘Horoscope effect’ not only for seasonal but also for non-seasonal allergens Clin. Exp. Allergy 1992 22 1003 1006 1468027 10.1111/j.1365-2222.1992.tb03028.x

[68] Soothill J Stokes C Turner M Norman A Taylor B Predisposing factors and the development of reaginic allergy in infancy Clin. Allergy 1976 6 305 319 963861 10.1111/j.1365-2222.1976.tb01911.x

[69] Korsgaard J Dahl R Sensitivity to house dust mite and grass pollen in adults: influence of the month of birth Clin. Allergy 1983 13 529 535 6640888 10.1111/j.1365-2222.1983.tb02634.x

[70] Sarpong S Karrison T Season of birth and cockroach allergen sensitization in children with asthma J. Allergy Clin. Immunol 1998 101 566 568 9564817 10.1016/S0091-6749(98)70369-0

[71] Lendor C Johnson A Perzanowski M Chew G Goldstein I Kelvin E Perera F Miller R Effects of winter birth season and prenatal cockroach and mouse allergen exposure on indoor allergen-specific cord blood mononuclear cell proliferation and cytokine production Ann. Allergy Asthma Immunol 2008 101 193 199 18727476 10.1016/S1081-1206(10)60209-8 PMC2582011

[72] Björkstén F Suoniemi I Koski V Neonatal birch-pollen contact and subsequent allergy to birch pollen Clin. Allergy 1980 10 585 591 7438417 10.1111/j.1365-2222.1980.tb02140.x

[73] Pearson D Freed D Taylor G Respiratory allergy and month of birth Clin. Allergy 1977 7 29 33 872354 10.1111/j.1365-2222.1977.tb01421.x

[74] Quoix E Bessot J Kopferschmitt-Kubler M Fraisse P Pauli G Positive skin tests to aero-allergens and month of birth Allergy 1988 43 127 131 3364625 10.1111/j.1398-9995.1988.tb00406.x

[75] Kemp A Relationship between the time of birth and the development of immediate hypersensitivity to grass-pollen antigens Med. J. Aust 1979 1 263 264 449781 10.5694/j.1326-5377.1979.tb112072.x

[76] Robert J Carron R Pollinose precoce des natifs du taureau (Early pollen allergy in children born in spring) Rev. Franc. Allergol 1979 19 153 155

[77] Pedersen P Weeke E Month of birth in asthma and allergic rhinitis Scand. J. Prim. Health Care 1983 1 97 101 6545046 10.3109/02813438309038476

[78] Croner S Kjellman N Predictors of atopic disease: cord blood IgE and month of birth Allergy 1986 41 68 70 3963316 10.1111/j.1398-9995.1986.tb00277.x

[79] Sarpong S Hamilton R Eggleston P Adkinson N Socioeconomic status and race as risk factors for cockroach allergen exposure and sensitization in children with asthma J. Allergy Clin. Immunol 1996 97 1393 1401 8648037 10.1016/s0091-6749(96)70209-9

[80] McDaniel M Paxson C Waldfogel J Racial disparities in childhood asthmas in the United States: evidence from the National Health Interview Survey, 1997 to 2003 Pediatrics 2006 117 e868 e877 16651291 10.1542/peds.2005-1721

[81] Litonjua A Carey V Weiss S Gold D Race, socioeconomic factors, and area of residence are associated with asthma prevalence Pediatr. Pulmonol 1999 28 394 401 10587412 10.1002/(sici)1099-0496(199912)28:6<394::aid-ppul2>3.0.co;2-6

[82] Frick O German D Mills J Development of allergy in children. I. Association with virus infections J. Allergy Clin. Immunol 1979 63 228 241 85648 10.1016/0091-6749(79)90106-4 PMC7133211

[83] Berg-Berkhoff G Schüz J Blettner M Münster E Schlaefer K Wahrendorf J Schlehofer B History of allergic disease and epilepsy and risk of glioma and meningioma (INTERPHONE study group, Germany) Eur. J. Epidemiol 2009 24 433 440 19484497 10.1007/s10654-009-9355-6

[84] Wigertz A Lönn S Schwartzbaum J Hall P Auvinen A Christensen H Johansen C Klæboe L Salminen T Schoemaker M Swerdlow A Tynes T Feychting M Allergic condition and brain tumor risk Am. J. Epidemiol 2007 166 941 950 17646205 10.1093/aje/kwm203

[85] Schwartzbaum J Jonsson F Ahlbom A Preston-Martin S Lönn S Söderberg K Feychting M Cohort studies of association between self-reported allergic conditions, immune-related diagnoses and glioma and meningioma risk Int. J. Cancer 2003 106 423 428 12845684 10.1002/ijc.11230

[86] Hochberg F Toniolo P Cole P Nonoccupational risk indicators of glioblastoma in adults J. Neurooncol 1990 8 55 60 2319291 10.1007/BF00182087

[87] Schlehofer B Blettner M Becker N Martinsohn C Wahrendorf J Medical risk factors and the development of brain tumors Cancer 1992 69 2541 2547 1568177 10.1002/1097-0142(19920515)69:10<2541::aid-cncr2820691025>3.0.co;2-h

[88] Schlehofer B Blettner M Preston-Martin S Niehoff D Wahrendorf J Arslan A Ahlbom A Choi W Giles G Howe G Little J Ménégoz F Ryan P Role of medical history in brain tumour development. Results from the international adult brain tumour study Int. J. Cancer 1999 82 155 160 10389745 10.1002/(sici)1097-0215(19990719)82:2<155::aid-ijc1>3.0.co;2-p

[89] Ryan P Lee M North B McMichael A Risk factors for tumors of the brain and meninges: results from the Adelaide Adult Brain Tumor Study Int. J. Cancer 1992 51 20 27 1563840 10.1002/ijc.2910510105

[90] Brenner A Linet M Fine H Shapiro W Selker R Black P Inskip P History of allergies and autoimmune diseases and risk of brain tumors in adults Int. J. Cancer 2002 99 252 259 11979441 10.1002/ijc.10320

[91] Linos E Raine T Alonso A Michaud D Atopy and risk of brain tumors: a meta-analysis. J Natl. Cancer Inst 2007 99 1544 1550 10.1093/jnci/djm170 17925535

[92] Wang H Diepgen T Is atopy a protective or a risk factor for cancer? A review of epidemiological studies Allergy 2005 60 1098 1111 16076292 10.1111/j.1398-9995.2005.00813.x

[93] Carrozzi L Viegi G Allergy and cancer: a biological and epidemiological rebus Allergy 2005 60 1095 1097 16076291 10.1111/j.1398-9995.2005.00940.x

[94] Hagströmer L Ye W Nyrén O Emtestam L Incidence of cancer among patients with atopic dermatitis Arch. Dermatol 2005 141 1123 1127 16172309 10.1001/archderm.141.9.1123

[95] Turner M Chen Y Krewski D Ghadirian P Thun M Calle E Cancer mortality among US men and women with asthma and hay fever Am. J. Epidemiol 2005 162 212 221 15987724 10.1093/aje/kwi193

[96] Siegmund B Schlehofer B Wahrendorf J Investigation on primary brain tumours and the co-morbidity with diabetes mellitus and atopic diseases in frame of the EPIC study Brain Tumor Epidemiology Consortium Annual Meeting Abstract Session, German Cancer Research Center, Heidelberg, Germany, 5–7 April, 2008.

[97] Eriksson N Mikoczy Z Hagmar L Cancer incidence in 13811 patients skin tested for allergy J. Investig. Allergol. Clin. Immunol 2005 15 161 166 16261950

[98] Cicuttini F Hurley S Forbes A Donnan G Salzberg M Giles G McNeil J Association of adult glioma with medical conditions, family and reproductive history Int. J. Cancer 1997 71 203 207 9139843 10.1002/(sici)1097-0215(19970410)71:2<203::aid-ijc13>3.0.co;2-i

[99] Dix A Brooks W Roszman T Morford L Immune defects observed in patients with primary malignant brain tumors J. Neuroimmunol 1999 100 216 232 10695732 10.1016/s0165-5728(99)00203-9

[100] Okada H Villa L Attanucci J Erff M Fellows W Lotze M Pollack I Chambers W Cytokine gene therapy of gliomas: effective induction of therapeutic immunity to intracranial tumors by peripheral immunization with interleukin-4 transduced glioma cells Gene Ther 2001 8 1157 1166 11509946 10.1038/sj.gt.3301496

[101] Benedetti S Di Meco F Cirenei N Bruzzone M Pollo B Florio N Caposio L Colombo M Cattaneo E Finocchiaro G IL-4 gene transfer for the treatment of experimental gliomas Adv. Exp. Med. Biol 1998 451 315 321 10026890 10.1007/978-1-4615-5357-1_49

[102] Alfven T Braun-Fahrländer C Brunekreef B von Mutius E Riedler J Scheynius A van Hage M Wickman M Benz M Buddle J Michels K Schram D Ublagger E Waser M Pershagen G the PARSIFAL study group Allergic diseases and atopic sensitization in children related to farming and anthroposophic lifestyle—the PARSIFAL study Allergy 2006 61 414 421 16512802 10.1111/j.1398-9995.2005.00939.x

[103] Ege M Frei R Bieli C Schram-Bijkerk D Waser M Benz M Weiss G Nyberg F van Hage M Pershagen G Brunekreef B Riedler J Lauener R Braun-Fahrländer C von Mutius E the PASIFAL Study team Not all farming environments protect against the development of asthma and wheeze in children J. Allergy Clin. Immunol 2007 119 1140 1147 17349684 10.1016/j.jaci.2007.01.037

[104] Riedler J Braun-Fahrländer C Eder W Schreuer M Waser M Maisch S Carr D Schierl R Nowak D von Mutius E the ALEX Study Team Exposure to farming in early life and development of asthma and allergy: a cross-sectional survey Lancet 2001 358 1129 1133 11597666 10.1016/S0140-6736(01)06252-3

[105] Braun-Fahrländer C Gassner M Grize L Neu U Sennhauser F Varonier H Vuille J Wäthrich B The SCARPOL Team Prevalence of hay fever and allergic sensitization in farmer’s children and their peers living in the same rural community Clin. Exp. Allergy 1999 29 28 34 10051699 10.1046/j.1365-2222.1999.00479.x

[106] Riedler J Eder W Oberfeld G Schreuer M Austrian children living on a farm have less hay fever, asthma and allergic sensitization Clin. Exp. Allergy 2000 30 194 200 10651771 10.1046/j.1365-2222.2000.00799.x

[107] von Ehrenstein O von Mutius E IIIi S Baumann I Böhm B von Kries R Reduced risk of hay fever and asthma among children of farmers Clin. Exp. Allergy 2000 30 187 193 10651770 10.1046/j.1365-2222.2000.00801.x

[108] Kilpelainen M Terho E Helenius H Koskenvuo M Farm environment in childhood prevents the development of allergies Clin. Exp. Allergy 2000 30 201 208 10651772 10.1046/j.1365-2222.2000.00800.x

[109] Klintberg B Berglung N Lilga G Wickman M van Hage-Hamsten M Fewer allergic respiratory disorders among farmers’ children in a closed birth cohort from Sweden Eur. Respir. J 2001 17 1151 1157 11491158 10.1183/09031936.01.00027301

[110] Ernst P Cormier Y Relative scarcity of asthma and atopy among rural adolescents raised on a farm Am. J. Respir. Crit. Care Med 2000 161 1563 1566 10806155 10.1164/ajrccm.161.5.9908119

[111] Remes S Iivanainen K Koskela H Pekkanen J Which factors explain the lower prevalence of atopy amongst farmers’ children? Clin. Exp. Allergy 2003 33 427 434 12680856 10.1046/j.1365-2222.2003.01566.x

[112] Leynaert B Neukirch C Jarvis D Chinn S Burney P Neukirch F on behalf of the European Community Respiratory Health Survey Does living on a farm during childhood protect against asthma, allergic rhinitis, and atopy in adulthood? Am. J. Respir. Crit. Care Med 2001 164 1829 1834 11734431 10.1164/ajrccm.164.10.2103137

[113] Gassner-Bachmann M Wüthrich B Farmers’ children suffer less from hay fever and asthma Dtsch. Med. Wochenschr 2000 125 924 931 10967955 10.1055/s-2000-6778

[114] Vercelli D Advances in asthma and allergy genetics in 2007 J. Allergy Clin. Immunol 2008 122 267 271 18619666 10.1016/j.jaci.2008.06.008

[115] Godfrey R Asthma and IgE levels in rural and urban communities of The Gambia Clin. Allergy 1975 5 201 207 1139767 10.1111/j.1365-2222.1975.tb01853.x

[116] Bråbäck L Does farming provide protection from asthma and allergies? Acta Paediatr 2002 91 1147 1149 12463307

[117] Bach B Mølhave L Indeklimasyndromet: the sick building syndrome Ugeskr. Laeg 1987 149 1012 1017 3576801

[118] Population and Public Health Branch (PPHB), Health Canada Farmers at lower risks for many diseases Farm Family Health 1995 3 1 3

[119] Blair A Sandler D Tarone R Lubin J Thomas K Hoppin J Samanic C Coble J Kamel F Knott C Dosemeci M Zahm S Lynch C Rothman N Alavanja M Mortality among participants in the agricultural health study Ann. Epidemiol 2005 15 279 285 15780775 10.1016/j.annepidem.2004.08.008

[120] Ronco G Costa G Lynge E Cancer risk among Danish and Italian farmers Br. J. Ind. Med 1992 49 220 225 1571291 10.1136/oem.49.4.220 PMC1012102

[121] Schoemaker M Swerdlow A Hepworth S van Tongeren M Muir K McKinney P History of allergies and risk of glioma in adults Int. J. Cancer 2006 119 2165 2172 16823851 10.1002/ijc.22091

[122] Goedert J Coté T Virgo P Scoppa S Kingma D Gail M Jaffe E Biggar R for the AIDS-Cancer Match Study Group Spectrum of AIDS-associated malignant disorders Lancet 1998 351 1833 1839 9652666 10.1016/s0140-6736(97)09028-4

[123] Frisch M Bigger R Engels E Goedert J for the AIDS-Cancer Match Registry Study Group Association of cancer with AIDS-related immunosuppression in adults JAMA 2001 285 1736 1745 11277828 10.1001/jama.285.13.1736

[124] Grulich A Wan X Law M Coates M Kaldor J Risk of cancer in people with AIDS AIDS 1999 13 839 843 10357384 10.1097/00002030-199905070-00014

[125] Schiff D Gliomas following organ transplantation: analysis of the contents of a tumor registry J. Neurosurg 2004 101 932 934 15597753 10.3171/jns.2004.101.6.0932

[126] Naumova E Mystery of seasonality: getting the rhythm of nature J. Public Health Policy 2006 27 2 12 16681184 10.1057/palgrave.jphp.3200061 PMC2483431

[127] Anderson J Seasonality of symptomatic bacterial urinary infections in women J. Epidemiol. Community Health 1983 37 286 290 6655418 10.1136/jech.37.4.286 PMC1052926

[128] Nagashima K Yasui K Kimura J Washizu M Yamaguchi K Mori W Induction of brain tumors by a newly isolated JC virus (Tokyo-1 strain) Am. J. Pathol 1984 116 455 463 6089567 PMC1900468

[129] Zu Rhein G Varakis J Perinatal induction of medulloblastomas in Syrian golden hamsters by a human polyoma virus (JC) Natl. Cancer Inst. Monogr 1979 51 205 208 225669

[130] London W Houff A Madden D Fuccillo D Gravell M Wallen W Palmer A Sever J Padgett B Walker D ZuRhein G Ohashi T Brain tumors in owl monkeys inoculated with a human polyomavirus (JC virus) Science 1978 201 1246 1249 211583 10.1126/science.211583

[131] London W Houff S McKeever P Wallen W Sever J Padgett B Walker D Viral induced astrocytomas in squirrel monkeys Prog. Clin. Biol. Res 1983 105 227 237 6304760

[132] Rollison D Helzlsouer K Alberg A Hoffman S Hou J Daniel R Shah K Major E Serum antibodies to JC virus, BK virus, simian 40 virus, and the risk of incident adult astrocytic brain tumors Cancer Epidemiol. Biomarkers Prev 2003 12 460 463 12750243

[133] Zimmerman H The significance of experimental gliomas for human disease Gliomas Current Concepts in Biology, Diagnosis, and Therapy Hekmatpanah J Springer-Verlag New York, NY, USA 1975 6 19

[134] Schuman L Choi N Gullen W Relationship of central nervous system neoplasms to Toxoplasma gondii infection Am. J. Public Health Nations Health 1967 57 848 856 6067209 10.2105/ajph.57.5.848 PMC1227362

[135] Wrensch M Weinberg A Wiencke J Miike R Sison J Wiemels J Barger G DeLorenze G Aldape K Kelsey K History of chickenpox and shingles and prevalence of antibodies to varicella-zoster virus and three other herpesviruses among adults with glioma and controls Am. J. Epidemiol 2005 161 929 938 15870157 10.1093/aje/kwi119

[136] Wrensch M Weinberg A Wiencke J Miike R Barger G Kelsey K Prevalence of antibodies to four herpesviruses among adults with glioma and controls Am. J. Epidemiol 2001 154 161 165 11447050 10.1093/aje/154.2.161

[137] Bithell J Draper G Gorbach P Association between malignant disease in children and maternal virus infections Br. Med. J 1973 24 706 708 4348514 10.1136/bmj.1.5855.706 PMC1588846

[138] Dickinson H Nyari T Parker L Childhood solid tumours in relation to infections in the community in Cumbria during pregnancy and around the time of birth Br. J. Cancer 2002 87 746 750 12232758 10.1038/sj.bjc.6600530 PMC2364254

[139] Linos A Kardara M Kosmidis H Katriou D Hatzis C Kontzoglou M Koumandakis E Tzartzatou-Stathopoulou F Reported influenza in pregnancy and childhood tumour Eur. J. Epidemiol 1998 14 471 475 9744679 10.1023/a:1007437200858

[140] Fear N Roman E Ansell P Bull D Malignant neoplasms of the brain during childhood: the role of prenatal and neonatal factors (United Kindom) Cancer Causes Control 2001 12 443 449 11545459 10.1023/a:1011201524589

[141] Linet M Gridley G Cnattingius S Nicholson H Martinsson U Glimelius B Adami H Zack M Maternal and perinatal risk factors for childhood brain tumors (Sweden) Cancer Causes Control 1996 7 437 448 8813432 10.1007/BF00052670

[142] Kaiser L Couch R Galasso G Glezen W Webster R Wright P Hayden F First International Symposium on Influenza and Other Respiratory Viruses: summary and overview: Kapalua, Maui, Hawaii, December 4–6, 1998 Antiviral Res 1999 42 149 175 10443529 10.1016/S0166-3542(99)00034-0 PMC7134157

[143] Melnikov O Nikolsky I Dugovskaya L Balitskaya N Kravchuk G Seasonal aspects of immunological reactivity of human and animal organism J. Hyg. Epidemiol. Microbiol. Immunol 1987 31 225 230 3611763

[144] Carandente F Angeli A De Vechi A Dammacco F Halberg F Multifrequency rhythms of immunologic functions Chronobiologia 1988 15 7 23 3046852

[145] Ko P Eyles D Burne T Mackay-Sim A McGrath J Season of birth and risk of brain tumors in adults Neurology 2005 64 1317 15824382 10.1212/wnl.64.7.1317

[146] Hillman L Haddad J Perinatal vitamin D metabolism. III. Factors influencing late gestational human serum 25-hydroxyvitamin D Am. J. Obstet. Gynecol 1976 125 196 200 1266899

[147] Verity C Burman D Beadle P Holton J Morris A Seasonal changes in perinatal vitamin D metabolism maternal and cord blood biochemistry in normal pregnancies Arch. Dis. Child 1981 56 943 948 7332342 10.1136/adc.56.12.943 PMC1627487

[148] Holick M Sunlight and vitamin D for bone and prevention of autoimmune diseases, cancers, and cardiovascular diseases Am. J. Clin. Nutr 2004 80 1678S 1688S 15585788 10.1093/ajcn/80.6.1678S

[149] Chatfield S Brand C Ebeling P Russell D Vitamin D deficiency in general medical inpatients in summer and winter Inter. Med. J 2007 37 377 382 10.1111/j.1445-5994.2007.01339.x 17535381

[150] Levis S Gomez A Jimenez C Veras L Ma F Lai S Hollis B Roos B Vitamin D deficiency and seasonal variation in an adult South Florida population J. Clin. Endocrinol. Metab 2005 90 1557 1562 15634725 10.1210/jc.2004-0746

[151] Eyles D Brown J Mackay-Sim A McGrath J Feron F Vitamin D3 and brain development Neuroscience 2003 118 641 653 12710973 10.1016/s0306-4522(03)00040-x

[152] Giovannucci E Expanding roles of vitamin D J. Clin. Endocrinol. Metab 2009 94 418 420 19193916 10.1210/jc.2008-2695

[153] Holick M Vitamin D: importance in the prevention of cancers, type 1 diabetes, heart disease, and osteoporosis Am. J. Clin. Nutr 2004 79 362 371 14985208 10.1093/ajcn/79.3.362

[154] Kricker A Armstrong B Does sunlight have a beneficial influence on certain cancers? Prog. Biophys. Mol. Biol 2006 92 132 139 16595142 10.1016/j.pbiomolbio.2006.02.015

[155] Giovannucci E The epidemiology of vitamin D and cancer incidence and mortality: a review (United States) Cancer Causes Control 2005 16 83 95 15868450 10.1007/s10552-004-1661-4

[156] McGrath J Keeping D Saha S Chant D Lieberman D O’Callaghan M Seasonal fluctuations in birth weight and neonatal limb length; does prenatal vitamin D influence neonatal size and shape? Early Hum. Dev 2005 81 609 618 15972254 10.1016/j.earlhumdev.2005.03.013

[157] Barker D Maternal nutrition, fetal nutrition, and disease in later life Nutrition 1997 13 807 813 9290095 10.1016/s0899-9007(97)00193-7

[158] Barker D The developmental origins of adult disease J. Am. Coll. Nutr 2004 23 588S 595S 15640511 10.1080/07315724.2004.10719428

[159] Bogovski P Bogovski S Animal species in which *N*-nitroso compounds induce cancer Int. J. Cancer 1981 27 471 474 7275353 10.1002/ijc.2910270408

[160] Koestner A Denlinger R Induction of neurogenic and lymphoid neoplasms by the feeding of threshold levels of methyl- and ethylnitrosourea precursors to adult rats Food Cosmet. Toxicol 1975 13 605 609 1205434 10.1016/0015-6264(75)90149-2

[161] Berleur M Cordier S The role of chemical, physical, or viral exposures and health factors in neurocarcinogenesis: Implications for epidemiologic studies of brain tumors Cancer Causes Control 1995 6 240 256 7612804 10.1007/BF00051796

[162] Boeing H Schlehofer B Blettner M Wahrendorf J Dietary carcinogens and the risk for glioma and meningioma in Germany Int. J. Cancer 1993 53 561 565 8436429 10.1002/ijc.2910530406

[163] Lee M Wrensch M Miike R Dietary and tobacco factors for adult onset glioma in the San Francisco Area (California, USA) Cancer Causes Control 1997 8 13 24 9051318 10.1023/a:1018470802969

[164] Rollison D Helzlsouer K Processed meat consumption and gliomas in a Maryland cohort Cancer Causes Control 2004 15 99 100 15049327 10.1023/b:caco.0000016675.85386.54

[165] Blowers L Preston-Martin S Mack W Dietary and other lifestyle factors of women with brain gliomas in Los Angeles County (California, USA) Cancer Causes Control 1997 8 5 12 9051317 10.1023/a:1018437031987

[166] Fraser P Chilvers C Fox J Risk assessment of N-nitroso compounds for human health: Human relevance, epidemiology and occupational exposure Oncology 1980 37 278 281 6255388 10.1159/000225452

[167] Terry M Howe G Pogoda J Zhang F Ahlbom A Choi W Giles G Little J Lubin F Ménégoz F Ryan P Schlehofer B Preston-Martin S An international case-control study of adult diet and brain tumor risk: a histology-specific analysis by food group Ann. Epidemiol 2009 19 161 171 19216998 10.1016/j.annepidem.2008.12.010 PMC3832293

[168] van Hanswijck de Jonge L Waller G Stettler N Ethnicity modifies seasonal variation in birth weight and weight gain of infants J. Nutr 2003 133 1415 1418 12730431 10.1093/jn/133.5.1415

[169] Buckles K Hungerman D Season of birth and later outcomes: old questions, new answers National Bureau of Economic Research Cambridge, MA, USA2008; Available online: http://www.nber.org/papers/w14573 (accessed on 08 April 2010).

[170] Chodick G Shalev V Goren I Inskip P Seasonality in birth weight in Israel: new evidence suggests several global patterns and different etiologies Ann. Epidemiol 2007 17 440 446 17300954 10.1016/j.annepidem.2006.10.013

[171] Curhan G Willett W Rimm E Spiegelman D Ascherio A Stampfer M Birth weight and adult hypertension, diabetes mellitus and obesity in US men Circulation 1996 94 3246 3250 8989136 10.1161/01.cir.94.12.3246

[172] Benson V Pirie K Green J Casabonne D Beral V Million Women Study Collaborators. Lifestyle factors and primary glioma and meningioma tumours in the Million Women Study cohort Br. J. Cancer 2008 99 185 190 18560401 10.1038/sj.bjc.6604445 PMC2453038

[173] Harder T Plagemann A Harder A Birth weight and subsequent risk of childhood primary brain tumors: a meta-analysis Am. J. Epidemiol 2008 168 366 373 18579539 10.1093/aje/kwn144

[174] Fallis G Hilditch J A comparison of seasonal variation in birthweights between rural Zaire and Ontario Can. J. Public Health 1989 80 205 208 2743244

[175] Powls A Botting N Cooke R Marlow N Handedness in very-low-birthweight (VLBW) children at 12 years of age: relation to perinatal and outcome variables Dev. Med. Child Neurol 1996 38 594 602 8674910 10.1111/j.1469-8749.1996.tb12124.x

[176] Saigal S Rosenbaum P Szatmari P Hoult L Non-right handedness among ELBW and term children at eight years in relation to cognitive function and school performance Dev. Med. Child Neurol 1992 34 425 433 1592195 10.1111/j.1469-8749.1992.tb11455.x

[177] Martin M Jones G Handedness and season of birth: a gender-invariant relation Cortex 1999 35 123 128 10213539 10.1016/s0010-9452(08)70790-1

[178] Inskip P Tarone R Brenner A Fine H Black P Shapiro W Selker R Linet M Handedness and risk of brain tumors in adults Cancer Epidemiol. Biomarkers Prev 2003 12 223 225 12646512

[179] Hepper P Shahidullah S White R Handedness in the human fetus Neuropsychologia 1991 29 1107 1111 1775228 10.1016/0028-3932(91)90080-r

[180] Geschwind N Galaburda A Cerebral Lateralization: Biological Mechanisms, Associations, and Pathology: I. A hypothesis and a program for research Arch. Neurol 1985 42 428 459 3994562 10.1001/archneur.1985.04060050026008

[181] Geschwind N Galaburda A Cerebral Lateralization: Biological Mechanisms, Associations, and Pathology: II. A hypothesis and a program for research Arch. Neurol 1985 42 521 552 3890812 10.1001/archneur.1985.04060060019009

[182] Geschwind N Galaburda A Cerebral Lateralization: Biological Mechanisms, Associations, and Pathology: III. A hypothesis and a program for research Arch. Neurol 1985 42 634 654 3874617 10.1001/archneur.1985.04060070024012

[183] Witelson S Neural sexual mosaicism: sexual differentiation of the human temporo-parietal region for functional asymmetry Psychoneuroendocrinology 1991 16 131 153 1961836 10.1016/0306-4530(91)90075-5

[184] Geschwind N Behan P Left-handedness: an association with immune disease, migraine, and developmental learning disorder Proc. Natl. Acad. Sci. U.S.A 1982 79 5097 5100 6956919 10.1073/pnas.79.16.5097 PMC346835

[185] Bryden M Mcmanus I Bulman-Fleming M Evaluating the empirical support for the Geschwing-Behan-Galaburda model of cerebral lateralization Brain Cogn 1994 26 103 167 7531983 10.1006/brcg.1994.1045

[186] Bryden P Bruyn J Fletcher P Handedness and health: an examination of the association between different handedness classifications and health disorders Laterality 2005 10 429 440 16191813 10.1080/13576500442000193

[187] Fritschi L Divitini M Talbot-Smith A Knuiman M Left-handedness and risk of breast cancer Br. J. Cancer 2007 97 686 687 17687338 10.1038/sj.bjc.6603920 PMC2360366

[188] Ramadhani M Elias S van Noord P Grobbee D Peeters P Uiterwaal C Innate left handedness and risk of breast cancer: case-cohort study BMJ 2005 331 882 883 16186135 10.1136/bmj.38572.440359.AE PMC1255796

[189] Titus-Ernstoff L Newcomb P Egan K Baron J Greenberg E Trichopoulos D Willett W Stampfer M Left-handedness in relation to breast cancer risk in postmenopausal women Epidemiology 2000 11 181 184 11021617 10.1097/00001648-200003000-00017

[190] Trichopoulos P Is breast cancer initiated in utero? Epidemiology 1990 1 95 96 2073510

[191] Nicholls M Seasonal trends in the birth of sinistrals Laterality 1998 3 241 253 15513087 10.1080/713754306

[192] Ronkainen H Pakarinen A Kirkinen P Kauppila A Physical exercise-induced changes and season-associated differences in the pituitary-ovarian function of runners and joggers J. Clin. Endocrinol. Metab 1985 60 416 422 3919040 10.1210/jcem-60-3-416

[193] Martikainen H Ruokonen A Tomás C Kauppila A Seasonal changes in pituitary function: amplification of midfollicular luteinizing hormone secretion during the dark season Fertil. Steril 1996 65 718 720 8654627 10.1016/s0015-0282(16)58202-8

[194] Testart J Frydman R Roger M Season influence of diurnal rhythms in the onset of the plasma luteinizing hormone surge in women J. Clin. Endocrinol. Metab 1982 55 374 377 7200993 10.1210/jcem-55-2-374

[195] Scheirs J Vingerhoets A Handedness and other laterality indices in women prenatally exposed to DES J. Clin. Exp. Neuropsychol 1995 17 725 730 8557813 10.1080/01688639508405162

[196] Schachter S Handedness in women with intrauterine exposure to diethylstilberstrol Neuropsychologia 1994 32 619 623 8084419 10.1016/0028-3932(94)90149-x

[197] Nass R Baker S Speiser P Virdis R Balsamo A Cacciari E Loche A Dumic M New M Hormones and handedness: left-hand bias in female congenital adrenal hyperplasia patients Neurology 1987 37 711 715 3561787 10.1212/wnl.37.4.711

[198] Stuerenburg H Breivik I Puchner M Lohmann F Kunze K Testosterone is locally metabolized in human brain tumors, with possible qualitative and quantitive changes in its effects Neuro. Endocrinol. Lett 1998 18 203 213

[199] Furth J Clifton K Experimental pituitary tumours The Pituitart Gland Hassis G Donovan B University of California Press Berkeley and Los Angeles, CA, USA 1966 460 497

[200] Avtsyn A Yablonovskaya L Effects of disturbances in the hormonal status on experimental brain tumors Acta Unio Int. Contra Cancrum 1964 20 1519 1522 14242944

[201] Collins R When left-handed mice live in right-handed worlds Science 1975 187 181 184 1111097 10.1126/science.1111097

[202] Vuoksimaa E Koskenvuo M Rose R Kaprio J Origins of handedness: a nationwide study of 30161 adults Neuropsychologia 2009 47 1294 1301 19428393 10.1016/j.neuropsychologia.2009.01.007 PMC2680751

[203] Medland S Duffy D Wright M Geffen G Hay D Levy F van-Beijsterveldt C Willemsen G Townsend G White V Hewitt A Mackey D Bailey J Slutske W Nyholt D Treloar S Martin N Boomsma D Genetic influences on handedness: data from 25,732 Australian and Dutch twin familes Neuropsychogia 2009 47 330 337 10.1016/j.neuropsychologia.2008.09.005 PMC2755095 18824185

[204] Derom C Thiery E Vlietinck R Loos R Derom R Handedness in twins according to zygosity and chorion type: a preliminary report Behav. Genet 1996 26 407 408 8771900 10.1007/BF02359484

[205] McManus I Handedness in twins: a critical review Neuropsychologia 1980 18 347 355 7191061 10.1016/0028-3932(80)90130-x

[206] Medland S Wright M Geffen G Hay D Levy F Martin N Duffy D Special twin environments, genetic influences and their effects on the handedness of twins and their siblings Twin Res 2003 6 119 130 12723998 10.1375/136905203321536245

[207] Sommer I Ramsey N Mandl R Kahn R Language lateralization in monozygotic twin pairs concordant and discordant for handedness Brain 2002 125 2710 2718 12429598 10.1093/brain/awf284

[208] Cosenza R Mingoti S Season of birth and handedness revisited Percept. Mot. Skills 1995 81 475 480 8570341 10.2466/pms.1995.81.2.475

[209] Ward J Milliken G Dodson D Stafford D Wallace M Handedness as a function of sex and age in a large population of Lemur J. Comp. Psychol 1990 104 167 173 2364661 10.1037/0735-7036.104.2.167

[210] Wells D Millsopp S Lateralized behaviour in the domestic cat Felis silvestris catus. Anim. Behav 2009 78 537 541 10.1037/a0026522 22201373

[211] Wells D Lateralised behaviour in the domestic dog Canis familiaris. Behav. Processes 2003 61 27 35 10.1016/s0376-6357(02)00161-4 12543480

[212] Hopkins W Bard K Hemispheric specialization in infant chimpanzees (Pan troglodytes), evidence for a relation with gender and arousal Dev. Psychobiol 1993 26 219 235 8354427 10.1002/dev.420260405

[213] Rothman K Greenland J Modern Epidemiology Lippincott-Raven Philadelphia, PA, USA 1998

[214] Hill A The environment and disease: Association or causation? Proc. R. Soc. Med 1965 58 295 300 14283879 10.1177/003591576505800503 PMC1898525

